# Supplementary material for: Validation of an omega-3 substrate challenge absorption test as an indicator of global fat lipolysis
Source: PLoS One. 2023 May 8;18(5):e0284651. doi: 10.1371/journal.pone.0284651 (PMC10166528; doi:10.1371/journal.pone.0284651)
Supplement: S1 File — (DOCX) [file pone.0284651.s002.docx]

Supplementary material: Ethical treatment of exocrine pancreatic insufficient pigs

1. Methods of anesthesia

Pigs were sedated using azaperone (Stresnil, ELANCO, Warsaw, Poland) at 4 mg/kg bw, i.m.. The pigs were then anaesthetised using 0.5–1.5% air mixture of Fluothane (Zeneca, Gothenburg, Sweden) and O_2_ as a carrier gas, at approximately 0.5–1 l/min in a close circuit respiratory system (Komesaroff Medical Developments, Melbourne, Australia). Surgical anaesthesia was indicated by the lack of a corneal reflex.

1. Methods to alleviate suffering.

Postoperative pain was prevented by the administration of buprenorphine (Temgesic®, Roche, Warsaw, Poland, 0.01 mg/kg bw, i.m.). Ampicillin (Ampicillin TZF, Polfa Tarchomin, Tarchomin, Polska) was administrated i.v. (15 mg/kg bw) for three days after surgery.

1. Methods of sacrifice

At the end of the study, all pigs were euthanized by an i.v. injection of an overdose of pentobarbital sodium (Euthanimal, Alfasan, Leżajsk, Polen, 100 mg/kg bw).
