# Supplementary material for: Validation of an omega-3 substrate challenge absorption test as an indicator of global fat lipolysis
Source: PLoS One. 2023 May 8;18(5):e0284651. doi: 10.1371/journal.pone.0284651 (PMC10166528; doi:10.1371/journal.pone.0284651)
Supplement: Attachment — Submitted filename: Reponse to Reviewers PONE-D-23-01728 revised.pdf [file pone.0284651.s004.pdf]

Jacobs School of Medicine
and Biomedical Sciences
University at Buffalo

March 23, 2023

To the Editors of PLoS ONE,

We appreciate your prompt review of our manuscript, "Validation of an omega-3 substrate challenge absorption test as an indicator of global fat lipolysis" (PONE-D-23-01728). Our responses to the Reviewers' comments are below in Calibri font.

Reviewer:

Under Abstract

The section is well written, conclusion is very much clear and concise

- Thank you.

Under Introduction

Comments

Overall the introduction is good and informative. I would only suggest to put some lines about the functional role of the omega-3 substrate challenge absorption test in this section rather than focusing more on the critical aspect of the coefficient of fat absorption (CFA) test.

- We have deleted some of the discussion of CFA and added a sentence to highlight the functional aspects of the omega-3 SACT.

Under Methodology

The methodology employed is robust, however information about following points need to be addressed:

- The methodology portion should be limited to the information about use of methods. You have added the background of exocrine pancreatic insufficient (EPI) pig model which is not needed here.
  - We deleted some of the more specific description of the EPI pig model.
- Give information about the following points:
  - Is the sample size sufficient?
    - The sample size was based on previous experiments using the EPI pig model and was estimated using G*Power software, version 3.1.9.7 for ANOVA with repeated measurements and within-between interactions, at α = 0:05 with 95% power, assuming *f* (effect size) = 1.2, for five study groups and five measurements, taking into consideration the correction for repeated measurements (0.5). The calculation yielded the total sample size of 10 animals and the 12 animals were taken considering the possible

risk of EPI surgery failure (1, 2, 3). This has been added to the Methods section.

- How many replications were performed?
  - Five replications were performed.
- Time of blood sampling?
  - As described in the Methods section in the last paragraph of the section sub-titled *"Exocrine pancreatic insufficient (EPI) pig model",* the timing of blood sampling was 0, 1, 2, 4, 6, 8, 12, and 24 hours. We added that the first (0) sampling was performed at 8:00 am local time.
- Sampling procedure, use of syringes and anticoagulant use is missing, time gap between blood sampling and analysis.
  - Blood samples were taken from the jugular vein catheter to 5 ml syringes (BD Syringe™, SKU 309646, BD Switzerland Sarl, 1262 Eysins, Vaud. Switzerland) and then transferred to BD Vacutainer® EDTA Tubes (SKU 366643, BD Switzerland Sarl, 1262 Eysins, Vaud. Switzerland). The collected blood was centrifuged at 4,000 rpm at 4°C for 10 min. Plasma was separated and stored at -80C. Plasma was analyzed within one month after study completion. This has been added to the Methods section.
- Features of the column use for fatty acid determination in GCMS.
  - Fatty acid analysis was carried out using a GC2010 Gas Chromatopgraph (Shimadzu) equipped with a SP2560 100-m fused silica capillary column. We have added this to the Methods section and provided a reference with the complete methodology (4).

Under statistical analysis

Robust

- We appreciate your external review.

Under results and discussion

Both the results are very informative. Well written.
No major changes need in this section.

- You should further relate your study with other such work being carried over recently.
  - We have added more description of the two relevant studies cited and how they relate to this work in the Discussion. We changed the order in which they are presented to improve the flow of theparagraph.
- Try to make this portion more coherent and improve the figure quality
  - We have expanded the sub-section title in the Results to make the work more coherent.
  - Revised Figures have been re-submitted as TIFF files.

All the authors appreciate the Reviewers' input and agree with these revisions to this submission. We hope you will accept these changes and consider this revised manuscript for publication in PLoS ONE.

Sincerely,

Drucy Borowitz, MD

Drucy Borowitz, MD
Corresponding author

1. Donaldson J, Fed'kiv O, Pawlowska M, Kowalik S, Erlwanger KH, Westrom B, et al. The effectiveness of enzymatic replacement therapy measured by turbidimetry and the lipaemic index in exocrine pancreatic insufficient young, growing pigs, fed a high-fat diet. Adv Med Sci. 2009;54(1):7-13.
2. Pierzynowska K, Valverde-Piedra J, Szymanczyk S, Prykhod'ko O, Pieszka M, Kardas M, et al. Pancreatic-like enzymes of microbial origin restore growth and normalize lipid absorption in a pig model with exocrine pancreatic insufficiency. Arch Med Sci. 2018;14(2):407-14.
3. Faul F, Erdfelder E, Lang AG, Buchner A. G*Power 3: a flexible statistical power analysis program for the social, behavioral, and biomedical sciences. Behav Res Methods. 2007;39(2):175-91.
4. Harris WS, Pottala JV, Vasan RS, Larson MG, Robins SJ. Changes in erythrocyte membrane trans and marine fatty acids between 1999 and 2006 in older Americans. J Nutr. 2012;142(7):1297-303.
