# Supplementary material for: Validation of an omega-3 substrate challenge absorption test as an indicator of global fat lipolysis
Source: PLoS One. 2023 May 8;18(5):e0284651. doi: 10.1371/journal.pone.0284651 (PMC10166528; doi:10.1371/journal.pone.0284651)
Supplement: Attachment — Submitted filename: Plos review.docx [file pone.0284651.s003.docx]

**Under Abstract**

The section is well written, conclusion is very much clear and concise

**Under Introduction**

Comments

Overall the introduction is good and informative. I would only suggest to put some lines about the functional role of the omega-3 substrate challenge absorption test in this section rather than focusing more on the critical aspect of the coefficient of fat absorption (CFA) test.

**Under Methodology**

The methodology employed is robust, however information about following points need to be addressed

The methodology portion should be limited to the information about use of methods. You have added the background of exocrine pancreatic insufficient (EPI) pig model which is not needed here. Give information about the following points

Is the sample size sufficient?

How many replications were performed?

Time of blood sampling?

Sampling procedure, use of syringes and anticoagulant use is missing, time gap between blood sampling and analysis.

Features of the column use for fatty acid determination in GCMS.

**Under statistical analysis**

Robust

**Under results and discussion**

Both the results are very informative. Well written.

No major changes need in this section. You should further relate your study with other such work being carried over recently.

Try to make this portion more coherent and improve the figure quality
